# Tannins in the Treatment of Diabetic Neuropathic Pain: Research Progress and Future Challenges

**DOI:** 10.3389/fphar.2021.805854

**Published:** 2022-01-10

**Authors:** Norsuhana Omar, Che Aishah Nazariah Ismail, Idris Long

**Affiliations:** ^1^ Department of Physiology, School of Medical Sciences, Health Campus, Universiti Sains Malaysia, Kubang Kerian, Malaysia; ^2^ Biomedical Science programme, School of Health Sciences, Health Campus, Universiti Sains Malaysia, Kubang Kerian, Malaysia

**Keywords:** diabetes mellitus, plant phytochemicals, tannins, diabetic neuropathic pain (DNP), preclinical

## Abstract

Diabetes mellitus and its consequences continue to put a significant demand on medical resources across the world. Diabetic neuropathic pain (DNP) is a frequent diabetes mellitus chronic microvascular outcome. Allodynia, hyperalgesia, and aberrant or lack of nerve fibre sensation are all symptoms of DNP. These clinical characteristics will lead to worse quality of life, sleep disruption, depression, and increased mortality. Although the availability of numerous medications that alleviate the symptoms of DNP, the lack of long-term efficacy and unfavourable side effects highlight the urgent need for novel treatment strategies. This review paper systematically analysed the preclinical research on the treatment of DNP using plant phytochemicals that contain only tannins. A total of 10 original articles involved in *in-vivo* and *in-vitro* experiments addressing the promising benefits of phytochemical tannins on DNP were examined between 2008 and 2021. The information given implies that these phytochemicals may have relevant pharmacological effects on DNP symptoms through their antihyperalgesic, anti-inflammatory, and antioxidant properties; however, because of the limited sample size and limitations of the studies conducted so far, we were unable to make definitive conclusions. Before tannins may be employed as therapeutic agents for DNP, more study is needed to establish the specific molecular mechanism for all of these activities along the pain pathway and examine the side effects of tannins in the treatment of DNP.

## Introduction

Diabetic neuropathic pain (DNP) is the most prevalent diabetes complication, affecting more than half of patients, and is associated with increased morbidity and mortality (Feldman et al., 2017). Tingling, burning, sharp, shooting, and lancinating, as well as electric shock sensations, are all symptoms of DNP. These signs lead to diminished daily routines, higher unemployment rates, sleep disruption, stress and mental health problems, physical co-morbidities, and even amputation (Gylfadottir et al., 2019). The pathogenesis of DNP is not fully understood. Several theories have been proposed to explain the pain associated with diabetic neuropathy, including changes in the blood vessels that supply the peripheral nerves; a neuroinflammation process accompanied by glial cell activation; changes in sodium and calcium channel expression; and, more recently, central pain mechanisms, including increased thalamic vascularity and an imbalance of the facilitatory/inhibitory pathways (Tesfaye et al., 2013).

The molecular mechanism of DNP might also be related to an imbalance in the generation of oxidative stress and antioxidant activity. Prolonged hyperglycemia causes glucotoxicity, which impairs several biological metabolome pathways such as the polyol, hexosamine, poly (ADP-ribose) polymerase (PARP), protein kinase C (PKC) and generation of the advanced glycation end product (AGE) (Ab Hamid et al., 2021). These processes result in the increased formation of free radicals such as hydrogen peroxide (H_2_O_2_), nitric oxide (NO), and superoxide anion (O2-), which cause cellular damage (Vincent et al., 2004). Hyperglycemia also activates inflammatory signalling pathways, which excrete a variety of mediators that aggravate the situation and contribute to the development of DNP. Following chronic hyperglycemia, the persistent generation of pro-inflammatory insults such as TNF-α and IL-1β, as well as oxidative stress markers, activates Toll-like receptors (TLRs) (Lawrence, 2009). As a result, these mechanisms translocate Nuclear Factor kappa-light-chain-enhancer β **(**NF-кβ) into nuclei and activate the expression of NF-кβ-dependent genes such pro-IL-1β, pro-IL-18, and Nod-like receptor protein 3 (NLRP3). Increased NF-кβ activation causes a rise in the creation of additional pro-inflammatory and immunological cells, such as T-cells, which further destroys the cells (Liu et al., 2017). Furthermore, a study has revealed that non-neuronal cells such as microglia and astrocytes have a role in the pathogenesis of DNP in a hyperglycemic environment (Wang et al., 2014). The glial-neuron crosstalk generates pathological pain in DNP, including allodynia and hyperalgesia, by releasing a variety of inflammatory mediators (Ismail et al., 2020).

Only three drugs are currently authorised in the United States by Food and Drug Administration (FDA) to treat DNP, which are duloxetine, a selective serotonin and norepinephrine reuptake inhibitor, pregabalin, an anticonvulsant, and tapentadol, a dual-action opioid receptor agonist and norepinephrine reuptake inhibitor (Freeman, 2013). All these treatments reduce pain by 30–50% but are limitedly prescribed because of their side effects. Therefore, natural products from plant secondary metabolites are now widely used to treat various chronic illnesses due to their low toxicity and high efficacy (Uddin et al., 2020).

Tannins are high-molecular-weight polyphenolic compounds found in a variety of plant species. Tannins bind to proteins and other chemical molecules, such as amino acids and alkaloids, and cause them to precipitate. The two most common tannin types are hydrolysable tannins and condensed tannins. Examples of hydrolysable tannins are gallic and ellagic acid. Whereas condensed tannins are gallocathecin, epigallocathecin, proanthocynidins and procynidin B2 (Laddha and Kulkarni, 2019). Tannins can be found in coffee, tea, wine, grapes, apricot, barley, peaches, dry fruits, mint, basil, rosemary, pomegranate, strawberries, amla, clove, rice, oat, rye, and other foods. Tannins are gaining popularity these days due to the health advantages linked with their antioxidant qualities (Ajebli and Eddouks, 2018).

Despite the health benefits of tannins, there are no systematic evaluations on tannins’ potential for treating DNP. Therefore, this study examined and synthesised research on tannins on DNP in *in-vivo* and *in-vitro* experiments to determine their antinociceptive effects in neuropathic pain models.

## Materials and Methods

For this systematic search, we developed a search strategy to identify relevant works of literature. This search strategy was limited to English articles. It used different combinations of the following keywords: neuropathic pain, plants metabolite, natural product, tannins, gallic acid, ellagic acid, epigallocatechin, and proanthocynidins in three databases: Scopus, PubMed, and Google Scholar.

**FIGURE 1:**
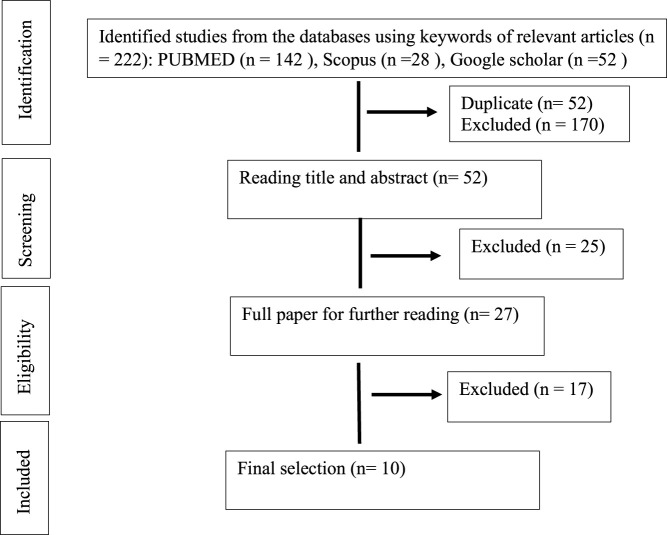
Search and selection results (PRISMA statement).

The databases were combed for studies that took place between 2008 and August 2021. Only DNP studies that included *in-vivo* and *in-vitro* experiments were included, as well as the use of compounds containing only phytochemical tannins (gallic acid, ellagic acid, epigallocatechin, and proanthocynidins) derived from medicinal plants for treatment. Studies were excluded according to the following exclusion criteria: studies in human beings and non-diabetic neuropathic pain, studies using polyphenol that contain another polyphenol such as coumarins and flavonoid, extracts or mixtures (as essential oils), review articles, meta-analyses, abstracts, conference proceedings, editorials/letters and case reports as shown in Figure 1 (PRISMA statement).

## Results

A total of 222 abstracts/citations were identified from the electronic search for preliminary review. After the removal of duplicates and screening for relevant titles and abstracts, a total of 27 articles were submitted for a full-text review. Ten articles fulfilled the inclusion and exclusion criteria established. In most articles analysed, the substances used were purchased commercially (70%). Only three studies were conducted as compounds isolated from plants (30%). Table 1 and Table 2 shows the summary of experimental studies using tannins phytochemical in DNP and composition of substances used.

**TABLE 1:** Characteristic of included studies.

Compound, plants species, source, concentration	Tannins constituents	Type of study, animals and diabetic model	Control group and duration of treatment	Daily dose (mg/kg) and routes of administration	Outcomes	References/country
Pure compound (Sigma Aldrich)	(−)‐epigallocatechin‐3‐O‐gallate	*In vivo* Male Swiss albino rats Single injection of STZ (55 mg/kg, i.p)	No treatment (−ve control) 5 weeks	25 mg/kg/orally/once/daily after fourth day diabetes induction	Reduce blood glucose	Egypt, (Abo-Salem et al., 2020)
					Increase Body weight	
					Improved serum lipids profile	
					Ameliorated plasma level of Nitric oxide (NO), IL‐6 and TNF‐α level	
					Reduced diabetes‐induced hyperalgesia in the behavioural tests (hot plate, formalin, tail immersion and carrageenan‐induced oedema model)	
Pure compound (Sigma Aldrich)	Catechin	*In vivo* Male Sprague Dawley rats. Single injection of STZ (55 mg/kg, i.p.)	No treatment (−ve control) 28 days	25 mg/kg and 50 mg/kg orally after 6 weeks of diabetes induction	Reduce blood glucose	India, (Addepalli and Suryavanshi, 2018)
					Increased body weight	
					Reduced Malondialdehyde (MDA)	
					Increased glutathione (GSH), catalase, Superoxide dismutase (SOD)	
					Reduced MMP-9	
Pure compound (Sigma Aldrich)	Proanthocyanidin B2	*In-vitro* Dorsal root ganglion neuron culture. Incubated with 45 mM high-glucose	Incubated in neurobasal medium (−ve control) 24 h	10 μg/ml	Decreased Neuronal ROS	China, (Zhang et al., 2018)
					Increased Neurite outgrowth	
					Decreased apoptosis	
					Increased cell viability increased GAP-43 mRNA	
Pure compound (Holliday and Co. Canada)	Epigallocatechin-gallate	*In vivo* Adult male Wistar rats. Intraperitoneal (i.p.) injection of STZ (60 mg/kg body weight	Injected with citrate buffer (−ve control) 10 weeks	2 g/L in drinking water	Not affected blood glucose level	Portugal, (Raposo et al., 2015)
					Not affected body weight	
					Reduced 8-OHdG immunoreaction	
					Reduced c-Fos IR neurons in the spinal cord	
					Amelioration of tactile allodynia and mechanical hyperalgesia	
Punica granatum L. (Lythraceae) extract (Ibn-Al-Nafess herbalist, Beirut, Lebanon)	gallic acid	*In vivo* Male Swiss-Webster mice. Alloxan (180 mg/kg) every 48 h for 3 time	Vehicle (0.9% sterile saline (−ve control) 1 day (acute) and 7 days (subacute)	25, 50, and 100 mg/kg, i.p after fourth day diabetes induction	Reduce blood glucose	Lebanon, (Raafat and Samy, 2014)
					Increase Body weight	
					Rise serum catalase activity	
					Improvement in hot plate latency	
					Improvement in tail-flick latency	
Pure compound Grape seed proanthocyanidins (Jianfeng Natural Product. Co. Ltd. (Tianjin China))	proanthocyanidins	*In vivo* Male Sprague–Dawley rats. Induced diabetes by 8 weeks of the high-carbohydrate/high fat diet and 2 injections of 25 mg/kg BW streptozotocin	Vehicle-treated (−ve control) 24 weeks	250 mg/kg by stomach tube	Blood glucose and body weight are not affected	China, (Ding et al., 2014)
					Increased the level of nerve conduction velocity (NCV)	
					Reduced the concentration of free Ca^2+^ and ER stress markers	
		*In vitro* RSC96 Schwann cells (SC) Challenged with DMEM containing 10% serum from diabetic rats	Cells treated with 10% serum from healthy rats (−ve control) 48 h	5, 10 and 20 μmol/L	Ameliorated cell injury	
					Decreased cytoplasmic free Ca^2+^	
					Alleviated ER stress	
					Decreased GRP78 and phospho-JNK expression	
*Vitis vinifera L.* (Vitaceae) grape seed extract	Proanthocyanidins	*In vivo* Male C57BL/6J mice. High Fat diet.	Normal diet (−ve control) 12 weeks	100 mg/kg and 250 mg/kg dissolved in drinking water, given orally once per day	Not reduced plasma blood glucose	Korea, (Jin et al., 2013)
					Reduced body weight gain	
					Increased IENF (intraepidermal innervation nerve fiber)	
*Cenostigma macrophyllum Tul.* (Fabaceae) Stem bark extracts	ellagic acid and valoneic acid dilactone	*In vivo* Male Wistar rats. streptozotocin (STZ, 40 mg/kg, i.v.)	No treatment (−ve control) STZ + insulin 2.5U (+ve control) 5 weeks	Ethanol extract (200 and 300 mg/kg, p.o.(chronic)	Not affected blood glucose and body weight	Brazil, (Piaulino et al., 2013)
				Ethyl acetate fraction (250 and 500 mg/kg, p.o.) after 28 days’ diabetic induction. (acute)	Increase the pain threshold	
Pure compound [Sigma (St. Louis, MO)]	Epigallocatechin-gallate	*In vivo* Male albino Wistar rats. Single injection of STZ (60 mg/kg, i.p.)	Normal saline (−ve control) 7 weeks	20 and 40 mg/kg orally after 1 week diabetes induction	Reduce blood glucose	Iran, (Baluchnejadmojarad and Roghani, 2012)
					Increased body weight lower nociceptive scores in both phases of the formalin test	
					Increased tail flick response latency Increased the vocalization threshold in Randall-Selitto test (mechanical hyperalgesia)	
					Reduced MDA, NO and increased SOD activity	
Pure compound (Jianfeng Natural Product. Co. Ltd. (Tianjin China))	Grape seed proanthocynidins	Male Wistar rat. Single injection of STZ (55 mg/kg, into tail vein)	Vehicle-treated (−ve control) 24 weeks	250 mg/kg/daily intragastric after 1 week diabetes induction	Reduced HbA1c and AGEs but not plasma blood glucose	China, (Cui et al., 2008)
					Increased body weight	
					Increased withdrawal threshold in von Frey test	
					Decreased MDA and increased SOD activity	

**TABLE 2:** The plants botanical and chemical composition.

Study	Compound, concentration	Source	Purity (%) (and grade if applicable)	Quality control reported? (Y/N)
(Abo-Salem et al., 2020)	Pure compound	Sigma Aldrich	≥90%	Y-HPLC
(Addepalli and Suryavanshi, 2018)	Pure compound	Sigma Aldrich	≥90%	Y-HPLC
(Zhang et al., 2018)	Pure compound	Sigma Aldrich	≥90%	Y-HPLC
(Raposo et al., 2015)	Pure compound	Holliday and co. Canada	≥90%	Y-HPLC
(Ding et al., 2014)	Pure compound	Jianfeng Natural Product. Co. Ltd. (Tianjin China)	≥90%	Y-HPLC
(Cui et al., 2008)	Pure compound	Jianfeng Natural Product. Co. Ltd. (Tianjin China)	≥90%	Y-HPLC and GCMS.
(Baluchnejadmojarad and Roghani, 2012)	Pure compound	Sigma (St. Louis, MO)	≥90%	Y-HPLC

Study	Species, source, concentration	Quality control reported? (Y/N)	Chemical analysis reported? (Y/N)
(Raafat and Samy, 2014)	Dried *Punica granatum L.* (Lythraceae) fruit, Ibn-Al-Nafess herbalist, Beirut, Lebanon	Y-Authenticated with reference sample and voucher specimen (PS-13–11) deposited in the faculty herbarium	Y-HPLC
(Jin et al., 2013)	*Vitis vinifera L.* (Vitaceae) grape seed extract, Korea Hanlim Co. (Seoul, Korea)	Y-Prepared according to this protocol. The seeds of ripe grape were separated from pulp and shade-dried. Then the seeds were crushed into a powder. Seed material (1 kg) was extracted with 300 ml distilled water and 200 ml acetone. Then the extract was filtered and concentrated to a volume of 100–150 ml. Chloroform was added and the filtrates were pooled and evaporated below 50°C in a vacuum drier to yield final preparation of 3.5 g	Y-HPLC
(Piaulino et al., 2013)	Stem bark of *Cenostigma macrophyllum Tul.* (Fabaceae) was collected surrounding the Federal University of Piaui, Teresina, Brazil	Y-Authenticated with voucher specimen (TEPB 10.374) deposited in Graziela Barroso herbarium of Federal University of Piaui, Teresina, Brazil	Y- Thin layer chromatography

## Discussion

Male rats from various species such as Swiss albino (Abo-Salem et al., 2020), Sprague-Dawley (Ding et al., 2014; Addepalli and Suryavanshi, 2018), Wistar (Cui et al., 2008; Piaulino et al., 2013; Raposo et al., 2015) and albino Wistar (Baluchnejadmojarad and Roghani, 2012) were used in the majority of the studies as DNP models. Two more studies used male Swiss Webster (Raafat and Samy, 2014) and C57BL/6J mice (Jin et al., 2013), while two investigations used cell cultures of dorsal root ganglion neuron (Zhang et al., 2018) and RSC96 Schwann cells (Ding et al., 2014) as a model for DNP. The most common technique for inducing diabetes and simulating the DNP is a single dose of streptozotocin (STZ) injection into the intraperitoneal and the tail vein. Other substances, such as alloxan, have also been used to cause diabetes. Mostly the rats or mice induced to be diabetic in these studies were type 1 diabetes. Diabetes can also be generated by changes in diet, such as a high carbohydrate and fat diet, which can lead to type 2 diabetes (Jin et al., 2013; Ding et al., 2014). However, we did not find a study to investigate the effects of phytochemical tannins on DNP using genetic modification in rats or mice.

Hyperalgesia and allodynia are symptoms of DNP linked to long-term hyperglycemia, insulin insufficiency or resistance, and dyslipidemia (Kim and Feldman, 2012). Many of the diabetes medications developed aimed to correct these problems and restore the situation by reducing the blood glucose level and increasing body weight. It has been demonstrated that taking (−) ‐Epigallocatechin‐3‐O‐gallate (EGCG) orally at a dose of 25 mg/kg for 5 weeks can lower serum blood glucose levels, improve serum lipid profiles, and increase body weight (Abo-Salem et al., 2020). Other studies were done by Addepalli and Suryavanshi (2018) used catechin, Raafat and Samy (2014) used *Punica granatum L.* (Lythraceae) extract, and Baluchnejadmojarad and Roghani (2012) used EGCG have validated the benefits of phytochemical tannins in lowering blood glucose and increasing body weight. However, other investigations using EGCC and stem bark extracts of *Cenostigma macrophyllum Tul* (Fabaceae) on male Wistar rats by Raposo et al. (2015) and Piaulino et al. (2013) failed to restore blood glucose levels and body weight gain. A study by Cui et al. (2008) used grape seed proanthocynidins on male Wistar rats and found that they could lower HbA1c and AGEs while increasing body weight but not blood glucose levels. In high glucose dorsal root ganglia (DRG) culture, administration of Proanthocyanidin B2 (10 μg/ml) restored the neurotoxic effect generated by glucose challenge (Zhang et al., 2018). The primary afferent neurons in the DRG are altered by incubated with high glucose concentration. This glucose challenge causes hyperglycemia, which inhibits neuronal development and causes oxidative stress and mitochondrial malfunction, leading to apoptotic cell death in DRG (Chowdhury et al., 2010; Akude et al., 2011).

Furthermore, phytochemical tannin treatment did not much affect blood glucose levels or body weight gain in a type 2 DNP animal model. Ding et al. (2014) observed that grape seed proanthocynidins did not affect blood glucose levels or body weight in male Sprague–Dawley rats fed with a high-carbohydrate, high-fat diet with two injections of 25 mg/kg of STZ. Jin et al. (2013) discovered that providing a high-fat diet to male C57BL/6J mice and given *Vitis vinifera L.* (Vitaceae) grape seed extract (VVE) did not lower blood glucose levels while simultaneously not increasing body weight. Insulin resistance and insulin insufficiency are two characteristics of type 2 diabetes. The natural mechanism of metabolic dysfunctions in human type 2 diabetes would be precisely mimicked by feeding the animal a high carbohydrate and high-fat diet followed by a low-dose STZ injection (Srinivasan et al., 2005). Insulin resistance, one of the critical characteristics of type 2 diabetes, is triggered by a high carbohydrate and fat diet. Low-dose STZ injections can cause a modest decrease in insulin production, similar to the latter stages of type 2 diabetes (Stott and Marino, 2020). However, the benefit of phytochemical tannin treatment on hyperglycemia and body weight in the DNP animal model is equivocal, with findings varying depending on the rat species, types of diabetes, DNP induction techniques, and substances delivery procedures.

Phytochemical tannins have been shown to have neuroprotective benefits in diabetic complications due to their anti-inflammatory and antioxidant characteristics (Meng et al., 2019). When EGCG was given orally at a 25 mg/kg dose for 5 weeks, inflammatory markers such as plasma IL-6, NO NO, and TNF-α were reduced (Abo-Salem et al., 2020). When given orally at dosages of 25 mg/kg and 50 mg/kg for 28 days, catechin can reduce matrix metalloproteinase-9 (MMP-9) levels (Addepalli and Suryavanshi, 2018). Raposo et al. (2015) discovered that EGCC at a dose of 2 g/L in drinking water for 10 weeks decreased Fos IR neurons in male Wistar rats, a marker for nociceptive response.

In addition to their anti-inflammatory properties, phytochemical tannins play a crucial role in preventing DNP because of their antioxidant activity. Catechin (25 mg/kg and 50 mg/kg orally for 28 days) decreased malondialdehyde (MDA) but raised the reduced glutathione (GSH), catalase, and superoxide dismutase (SOD) levels in STZ-induced diabetic rats (Addepalli and Suryavanshi, 2018). Baluchnejadmojarad and Roghani (2012) and Cui et al. (2008) investigations also revealed comparable impact. In STZ-induced diabetes, in male albino Wistar rats, treatment with EGCG (20 and 40 mg/kg BW orally) for 7 weeks decreased blood MDA and NO levels while increasing SOD level (Baluchnejadmojarad and Roghani, 2012). In STZ-induced diabetic male Wistar rats, grape seed proanthocynidins (250 mg/kg/daily intragastric) therapy for 24 weeks decreased plasma MDA and elevated SOD levels (Cui et al., 2008). Raposo et al. (2015) found that early treatment with EGCG decreased the 8-hydroxy-21-deoxyguanosine (8-OHdG) immunoreaction, preventing oxidative stress damage (Raposo et al., 2015).

In tunicamycin-treated sciatic nerves and rat Schwann cells (RSC cells), administration of proanthocyanidin and its metabolites catechin and epicatechin decreased cell damage. It downregulated the expression level of endoplasmic reticulum (ER) stress proteins (Ding et al., 2014). Schwann cells (SC) create lipid-rich myelin sheaths to extend their plasma membrane to conduct the action potential along axons and maintain axonal integrity (Eckersley, 2002). SC must generate many myelin membrane proteins, cholesterol, and membrane lipids through the secretory route during the active phase of myelination (Clague and Hammond, 2006). Demyelination and axonal degeneration, which are early stages of DNP, can be caused by disruptions in the secretory system, dependent on ER homeostasis (Lin and Popko, 2009).

Treatment with phytochemical tannins has also been shown to help with DNP hyperalgesia and allodynia symptoms. Its anti-inflammatory, antioxidant, and antihyperalgesic properties are thought to be the mechanism for decreasing these symptoms (Stott and Marino, 2020). According to Abo-Salem et al. (2020), treatment with EGCG alleviated hyperalgesia responses indicated by a hot plate, tail immersion, formalin, and carrageenan-induced oedema tests in STZ-induced diabetic rats. After being treated with EGCG for 10 weeks, STZ-induced diabetics also had a higher paw withdrawal threshold (PWT), indicating that tactile allodynia and mechanical hyperalgesia had improved (Raposo et al., 2015). In diabetic rats, chronic EGCG therapy (40 mg/kg) dramatically reduced hyperalgesia (formalin test, hot tail immersion test, and paw pressure test) as compared to untreated diabetics (Baluchnejadmojarad and Roghani, 2012). In the hot plate and tail-flick tests, *Punica granatum L.* (Lythraceae) extract significantly reduced thermal and tail-flick latency in alloxan-induced diabetic mice (Raafat and Samy, 2014). Piaulino et al. (2013) found that treating diabetic Wistar rats with stem bark extracts of *Cenostigma macrophyllum Tul.*(Fabaceae) raised mechanical nociceptive threshold (MNT) by using von Frey filaments as compared to untreated diabetic rats. In diabetic rats, grape seed proanthocyanidin extract can also reduce mechanical allodynia by the von Frey test (Cui et al., 2008).

## Strength and Limitation

This study offers a thorough evaluation of research progress on tannins and their efficacy in the treatment of symptomatic DNP. Tannins have been shown to alleviate the hyperalgesia and allodynia symptoms associated with DNP through their anti-inflammatory, antioxidant, and antihyperalgesic properties. We discovered the most relevant articles since we took a thorough approach with all search keywords. This study can be used as a baseline to offer information regarding the efficacy of tannins as a therapeutic therapy for symptomatic DNP. Phytochemical tannins have anti-inflammatory, antioxidant, and antihyperalgesic properties to help with the hyperalgesia and allodynia symptoms of DNP. However, we could not draw any definitive findings because of the small sample size. Attempts to re-investigate also fell short of including new tannins and DNP treatment data.

There are limitations to the research conducted thus far to examine the potential of tannins in DNP. The drawback of the tannins studies and their efficacy on symptomatic DNP was that some of the models employed did not entirely replicate the DNP condition. Zhang et al. (2018) employed DRG culture in high glucose conditions, which did not accurately imitate the situation of DNP *in vivo*. *In-vivo* trials, on the other hand, lacked a control group that was treated with current medications authorised to treat symptomatic DNP for comparison. Many research intended to lower blood glucose levels and increase body weight, primarily the type 1 diabetes model but did not compare their findings to those treated with metformin and insulin. Metformin and insulin are the medication taken by the diabetic patient to control their glucose level in the blood. Tannins anti-inflammatory, antioxidant, and antihyperalgesic properties are also not comparable to those of other approved drugs such as duloxetine, pregabalin, and tapentadol (Freeman, 2013).

Tannins research as a possible symptomatic DNP therapy has yet to identify a specific molecular basis for its anti-inflammatory, antioxidant, and antihyperalgesic properties. Only Zhang et al. (2018) proposed that the PI3K/Akt signalling pathway was engaged in the neurotoxicity of high glucose conditions in DRG culture. Furthermore, most research concentrates exclusively on behaviour analysis and changes in inflammatory markers, with a little examination into molecular changes in the expression of specific genes, proteins, and receptors along the pain pathway, which begins in the peripheral nervous system and ends in the brain. There is no evidence on whether tannins can modify these specific genes, proteins, and receptors to decrease DNP symptoms. Furthermore, no toxicological studies have been conducted to examine the toxicities of tannins on liver and kidney biochemical markers.

## Priorities for Future Research

A future investigation on tannins and their efficacy on DNP symptoms should reveal a precise molecular mechanism for their anti-inflammatory, antioxidant, and antihyperalgesic effects along the pain pathway. To investigate the effectiveness of tannins, a suitable *in vitro* and *in vivo* model to simulate the situation at the periphery, spinal cord, and brain during DNP and changes in particular genes, proteins, and receptors when treated with tannins, is required. In treating DNP symptoms, a specific molecular pathway connected to anti-inflammatory, antioxidant, and antihyperalgesic properties of tannins must be targeted and investigated to elucidate its molecular function better. A comparison of the effects of tannins and approved drugs on DNP symptoms is also required to see whether tannins’ results are equal or even superior to all of these treatments. Because the aetiology of DNP includes multiple elements and pathways, a combination of medications may be effective.

## Conclusion

Tannins, a phytochemical, help to alleviate DNP symptoms. These results are due to its hypoglycaemic effect, most visible in type 1 diabetic rats but not in type 2 diabetic rats. Phytochemical tannins lower blood glucose levels while increasing body weight in insulin insufficiency (type 1 diabetes), but not in insulin resistance or hyperlipidaemia studies (type 2 diabetes). Phytochemical tannins can be used as an anti-inflammatory, antioxidant, and antihyperalgesic to help with the hyperalgesia and allodynia symptoms of DNP. Tannins, a phytochemical, might potentially be used to treat DNP. However, we could not make definitive conclusions because of the limited sample size and limitations of the studies conducted. Before tannins may be employed as therapeutic agents for DNP, more study is needed to establish the specific molecular mechanism for all of these tannins properties along the pain pathway and examine the side effects of tannins in the treatment of DNP.

## Data Availability

The original contributions presented in the study are included in the article/Supplementary Materials, further inquiries can be directed to the corresponding author.
